# Porcine reproductive and respiratory syndrome virus inhibits MARC-145 proliferation via inducing apoptosis and G2/M arrest by activation of Chk/Cdc25C and p53/p21 pathway

**DOI:** 10.1186/s12985-018-1081-9

**Published:** 2018-11-06

**Authors:** Linlin Song, Ximeng Han, Cunyu Jia, Xin Zhang, Yunjie Jiao, Taofeng Du, Shuqi Xiao, Julian A. Hiscox, En-Min Zhou, Yang Mu

**Affiliations:** 1 Department of Preventive Veterinary Medicine, College of Veterinary Medicine, Northwest A&F University, Yangling, Shaanxi China; 2 Scientific Observing and Experimental Station of Veterinary Pharmacology and Diagnostic Technology, Ministry of Agriculture, Yangling, Shaanxi China; 3 Department of Infection Biology, Institute of Infection and Global Health, University of Liverpool, Liverpool, UK

**Keywords:** Porcine reproductive and respiratory syndrome virus, G2/M arrest, Cdc2-cyclinB1 complex, Cdc25C, p53

## Abstract

Porcine reproductive and respiratory syndrome virus(PRRSV) is an important immunosuppressive virus which can suppresses infected cells proliferation. In this work, we examined PRRSV ability to manipulate cell cycle progression of MARC-145 cells and explored the potential molecular mechanisms. The results showed that PRRSV infection imposed a growth-inhibitory effect on MARC-145 cells by inducing cell cycle arrest at G2/M phase. This arrest was due to the significant decrease of Cdc2-cyclinB1 complex activity in PRRSV-infected cells and the activity reduction was a result of Cdc2 Tyr15 phosphorylation and the accumulation of Cdc2 and cyclinB1 in the nucleus. Not only elevated Wee1 and Myt1 expression and inactivated Cdc25C, but also increase of p21 and 14–3-3σ in a p53-dependent manner caused the inhibitory Tyr15 phosphorylation of Cdc2. PRRSV infection also activated Chk1. Our data suggest PRRSV infection induces G2/M arrest via various molecular regulatory mechanisms. These results provide a new insights for PRRSV pathogenesis.

## Background

Porcine reproductive and respiratory syndrome (PRRS) is a detrimental disease in swine that was first recognized independently in North America in 1987 and in Europe in 1990. Since then, it has disseminated throughout the world and has caused significant morbidity and large economic losses of domestic swine [1, 2]. PRRSV belongs to the family of the *Arteriviridae,* order *Nidovirales*, and is a single-stranded, positive-sense RNA virus. In vivo, PRRSV infects subsets of pig macrophages that are mainly present in lungs and lymphoid organs. In vitro, PRRSV can infect primary cell cultures of porcine alveolar macrophages(PAMs), monocyte-derived macrophages, and monocyte-derived dendritic cells. Apart from primary cell cultures, PRRSV also can be cultivated in a few monkey kidney cell lines, such as MA-104, its derived MARC-145 cell lines, and infected and replicated in SJPL cell line [3]. Although PRRSV 1 and PRRSV 2 [4] have great differences in genomic nucleotide sequences and amino acid sequences of the open reading frame (ORF) regions, they are associated with different types of pathogenesis.

Apoptosis is one of the main types of programmed cell death, which involves a series of biochemical events leading to specific cellular morphologic characteristics and ultimate cell death. Numerous studies have suggested that PRRSV infection can induce cell apoptosis both in vitro and in vivo and that the induction mechanism is related with virus pathogenesis [5]. In a study performed on experimentally-infected pigs, PRRSV 2 infection was found to induce B- and T-cell apoptosis in areas of lymphoid organs [6]. PRRSV also causes apoptosis in infected macrophages and surrounding cells at the last stage of gestation during its replication in fetal implantation sites [7].

The life cycle of a dividing cell can be split into four stages: G1, S, G2 and mitosis(M), with cells that are no longer cycling being said to be quiescent or in G0. The two gap phases, G1 and G2, separate S phase, during which the DNA is replicated, and mitosis, in which it is divided between two new nuclei. After mitosis, the cell itself divides and each daughter cell begins the cycle again from G1, or exits the cell cycle into G0. Progression from one stage to the next is controlled by the activities of kinase complexes made up of cyclins bound to cyclin-dependent kinases(Cdk) and cell cycle checkpoints are important control mechanisms that ensure the proper execution of cell cycle events [8]. When DNA damage response occurs, the G2/M checkpoint blocks the entry into mitosis to allow damage repair or direct cell apoptosis. Numerous studies have suggested that many viruses and their related proteins can perturb the cell cycle and induce cell cycle arrest during infection [9, 10]. Although many studies have reported the pathogenic mechanisms of PRRSV infection, its effect on the cell cycle and the corresponding molecular mechanism have not been reported.

In this Study, we observed the effect of PRRSV infection on MARC-145 cells cycle and found that PRRSV infection promoted cell cycle arrest at G2/M phase. This cell cycle arrest was accompanied by inhibition of Cdc2-cyclinB1 kinase activity and a significant increase of phosphorylated Cdc2 at the Tyr15 inhibitory site. As far as we know, this is the first report that the G2/M arrest and reduced Cdc2-cyclinB1 activity induced by PRRSV infection involving activation of the Chk/Cdc25C and p53/p21 pathways, as well as elevating Myt1 and Wee1 expression.

## Materials and methods

### Cells and viruses

MARC-145 cells, a subclone of African green monkey kidney-derived MA-104 cells, were purchased from the China Center for Type Culture Collection(Wuhan, China). Cells were cultured either in 6-well plates or flasks, according to the standard culturing procedure with Dulbecco’s modified eagle medium(DMEM, ThermoFisher, #12800017) plus 10% fetal bovine serum(FBS), 100 μg/mL streptomycin, and 100 U/mL penicillin(Sigma-Aldrich, MO, USA) at 37 °C with 5% CO_2_. PRRSV 2 strains, SD16 (GenBank ID:JX087437.1), VR2332(GenBank ID:EF536003.1), CH-1a(GenBank ID: AY032626) and PRRSV 1 strain, GZ11-G1(GenBank ID:KF001144.1), were propagated and titrated in MARC-145 cells.

### Antibodies and reagents

p53 antibody(#9282), Phospho-p53(Ser15) antibody(#9284), p21^Walf1/Cip1^(12D1) rabbit mAb(#2947), cyclinB1 antibody(#4138), Cdc2(POH1) mouse mAb(#9116), phospho-Cdc2(Tyr15) (10A11) rabbit mAb(#4539), Cdc25C(5H9) rabbit mAb(#4688), phospho-Cdc25C(Ser216) (63F9) rabbit mAb(#4901), Myt1 antibody(#4282), Weel antibody(#4963), GAPDH(14C10) Rabbit mAb(#2118), and Alexa Fluor® 488 Phalloidin (#8878) (Phalloidin belongs to a class of toxins called phallotoxins. It functions by binding and stabilizing filamentous actin (F-actin) and effectively prevents the depolymerization of actin fibers. The properties of phalloidin make it a useful tool for investigating the distribution of F-actin in cells by labeling phalloidin with fluorescent analogs and using them to stain F-actin for light microscopy.) were all purchased from Cell Signaling Technology, Inc.(Danvers, MA, USA). Nocodazole(Nocodazole is a commonly used mitotic inhibitor which interferes with microtubule assembly thus interfering with mitosis duo to formation of multipolar spindles and leading to cell cycle arrest in G2/M [11]), mouse monoclonal anti-α-tubulin antibody, propidium iodide (PI), and Ribonuclease A (RNase A) were obtained from Sigma-Aldrich(St Louis, MO, USA). Mouse monoclonal anti-nucleocapsid(N) antibody(6D10) was previously made in our laboratory. Peroxidase-conjugated affinipure goat anti-mouse IgG(H + L), peroxidase-conjugated affinipure goat anti-rabbit IgG(H + L), and Cy™3-conjugated affiniPure goat anti-rabbit IgG(H + L) were purchased from Jackson ImmunoResearch Laboratories, Inc.(West Grove, PA, USA). Cell counting kit-8(CCK-8) was purchased from Beyotime Institute of Biotechnology(Shanghai, China). Alexa Fluor® 488 annexin V/Dead cell apoptosis kit was purchased from Invitrogen™ Life Technology(Grand Island, USA). DAPI, Dynabeads Protein G, FBS, and DMEM were purchased from ThermoFisher(Waltham, MA, USA).

### Determination of optimal inoculation

To determine the optimal inoculation, the standard curve of the absorbance of 450 nm (OD450nm) and cell number was obtained following the instructions of cell counting Kit-8 (CCK-8). MARC-145 cells were seeded in 96-well plates with 1 × 10^4^ cells/well and cultured to reach approximately 80% confluence at 37 °C, 5% CO_2_. Then, the cells were either mock-infected or infected with PRRSV SD16 at 0.01, 0.1, 1, or 5 multiplicity of infection (MOI) and reduplicated in 6 wells for every infective dose. At 0, 6, 12, 24, 36, 48, and 72 h after PRRSV infection, 10 μL CCK-8 solution was added, incubated for another 2 h, then OD450nm was measured with a Micro-Volume Spectrophotometer System(Epoch, BioTek Vermont, USA).

### Annexin V PI staining

MARC-145 cells were seeded in 6-well plates with 2 × 10^5^ cells/well and cultured to reach approximately 80% confluence. The cells were either mock-infected or infected with PRRSV SD16 at 1 MOI. At 0, 12, 24, 36, and 48 h after PRRSV infection, cells were collected and washed with cold phosphate-buffered saline(PBS). Cells were then resuspended in 1×annexin-binding buffer, followed by addition of Alexa Fluor® 488 annexin V and PI working solutions according to the manufacturer’s instructions. The apoptotic cells were analyzed by flow cytometry(Beckman Coulter Cytomics Altra, Brea, CA, USA).

### Cell cycle analysis

The cell cycle and nuclear DNA content were determined using PI staining and flow cytometry. Mock-infected or PRRSV-infected MARC-145 cells were collected, washed with PBS, and fixed with 70% cold ethanol. The cell pellets were resuspended in 1 mL PI solution containing 100 μg/mL PI, 100 μg/mL RNase A, and 0.1% Triton X-100 and incubated for 30 min at 4 °C. The DNA content was analyzed by flow cytometry(Beckman Coulter Cytomics Altra, Brea, CA, USA).

### Indirect immunofluorescence assay (IFA)

Mock-infected or PRRSV-infected MARC-145 cells were fixed with 4% paraformaldehyde for 10 min at RT, washed with PBS, permeabilized with 0.3% triton X-100/PBS for 3 min, then washed and blocked with 5% BSA/PBS. After washing, the cells were incubated with primary antibodies for 1 h at 37 °C, washed with PBS, and incubated with the corresponding secondary antibody. Finally, cells were stained with DAPI and visualized using Leica microsystems(Leica AF6000, Germany).

### Western blot analysis

Cells mock-infected and PRRSV-infected were harvested using Trypsin-EDTA(0.25%) (ThermoFisher, USA) digestion. After washing with PBS, cell samples were treated with NP40 lysis buffer (Beyotime, China), and then protein concentrations were determined using the Pierce BCA protein assay kit(ThermoFisher, USA). Equal amounts of protein were loaded and subjected to sodium dodecyl sulfate-polyacrylamide gel electrophoresis(SDS-PAGE) and then transferred to PVDF membranes(Millipore, USA) using BIO-RAD Mini Trans-blot. The membranes were blocked with 5% non-fat dry milk and then incubated with indicated primary antibodies overnight at 4 °C, followed by HRP-conjugated secondary antibodies. α-tubulin or GAPDH were used as loading control, and nocodazole-treated cells were used as positive control. The protein bands were visualized using ChemiDoc™ MP Imaging System(Bio-Rad,USA).

### Immunoprecipitation

p21 and Cdc2-cyclinB1 interactions were analyzed using immunoprecipitation according to introduction of the Dynabeads Protein G. Cells of mock-infected, PRRSV-infected, or nocodazole-treated were lysed using ice-cold NP40 cell lysis buffer, and the supernatants were obtained by centrifugation. Dynabeads-Ab complex was prepared by incubating Cdc2 mouse mAb with Dynabeads Protein G using a Catch and Release(version 2.0) reversible immunoprecipitation system(ThermoFisher, USA). Then, the supernatants were added to the tubes containing Dynabeads-Ab complex and incubated overnight at 4 °C. After washing with PBS, p21^Walf1/Cip1^ rabbit mAb and cyclinB1 antibody were used to detect the Dynabeads-Ab-Ag complex by western blot.

### Effect of PRRSV 1 and 2 strains infection on cyclinB1 and p-Cdc2 (Tyr15) expression analysis

MARC-145 cells were seeded in 6-well plates at a density of 2 × 10^5^ cells/well, and cultured to reach approximately 80% confluence. PRRSV strains SD16, GZ11-G1, VR-2332, or CH-1a were used to infect the cells at 1 MOI. At 48 h after viral infection, cells were collected and cyclinB1 and p-Cdc2 (Tyr15) expression were analyzed by western blot.

### Statistical analysis

Unless otherwise indicated, all data are shown as mean ± SEM of independent experiments performed in triplicate. GraphPad prism 6 was used for statistical analysis. Comparisons between groups were considered statistically significant at *p < 0.05*.

## Results

### PRRSV infection reduces number of MARC-145 cells

**Fig. 1:**
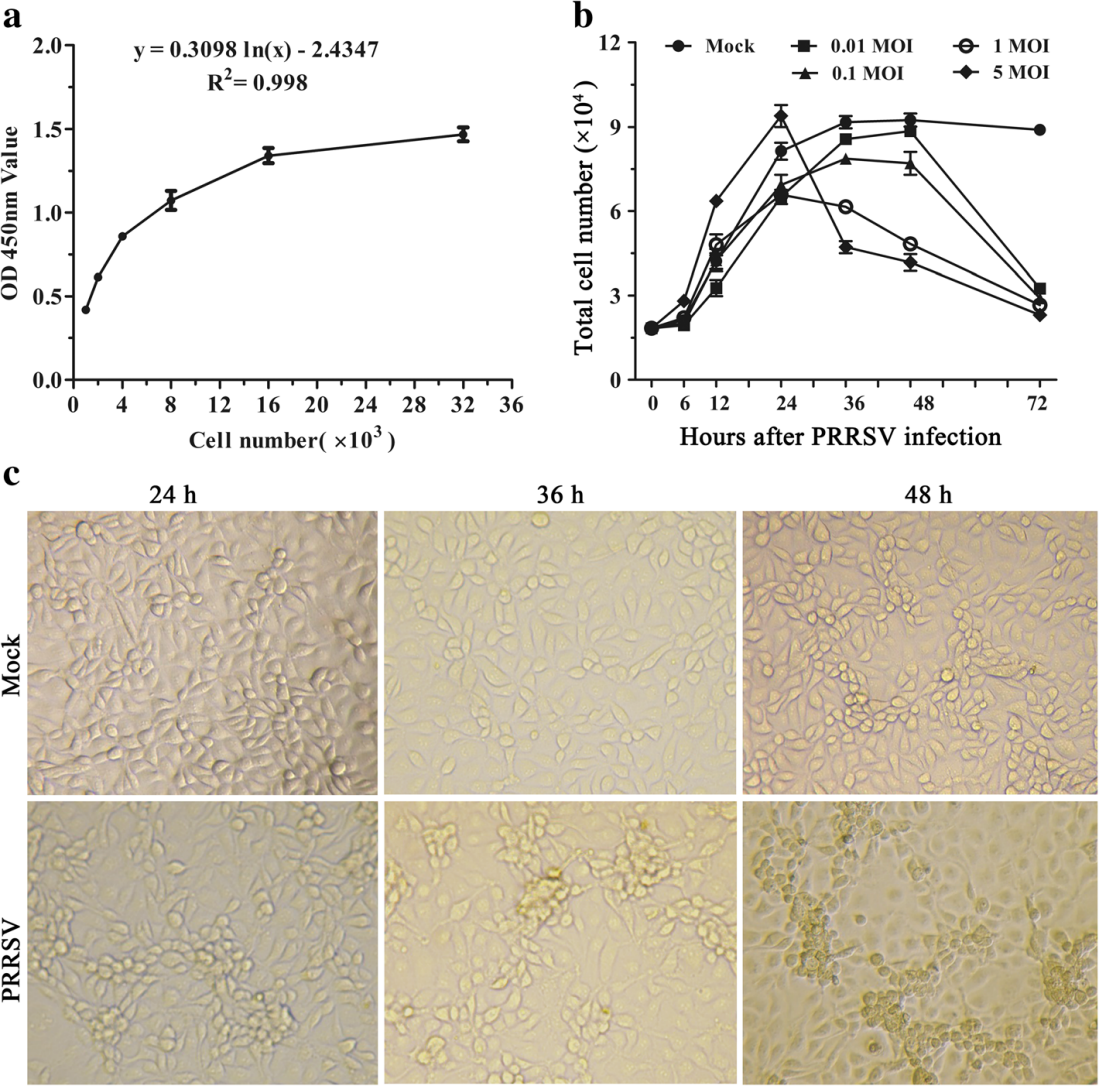
PRRSV infection causes reduction of MARC-145 cells number. **a**The standard curve of OD450nm and cell numbers. 1000, 2000, 4000, 8000, 16000, or 32000 cells were seeded in 96-well plate, and CCK-8 solution was added. The OD450nm were measured at 2 h after adding CCK-8 solution. Data shown are mean ± SEM from six repeated experiments. **b** Different MOI viral infection reduces MARC-145 cells number. MARC-145 cells were infected with PRRSV SD16 at 0.01, 0.1, 1, and 5 MOI. At 6, 12, 24, 36, 48, and 72 h after infection, CCK-8 solution was added, and the numbers of cells were calculated according to the standard curve. **c** PRRSV infection gives rise to typical CPE in infected MARC-145 cells (× 100)

Although PAM is the primary target cell of PRRSV, it is a terminally differentiated cell and can not divide and proliferate. So MARC-145 cell line was used in the presented study. To determine the optimal infective dose, a standard curve of OD450nm and cell number was produced according to CCK-8 instructions. The OD450nm was detected every half hour for 4 h after CCK-8 solution was added. The results indicate that the optimal time for detection is 2 h after adding CCK-8 solution. The slope equation “*y* = 0.3098 ln(*x*)-2.4347” was generated with *R*^2^ = 0.998 (Fig. 1a). The equation was used to determine the numbers of normal MARC-145 cells and PRRSV-infected MARC-145 cells with 0.01, 0.1, 1, and 5 MOI at 6, 12, 24, 36, 48, and 72 h after infection where x and y are cell number and OD450nm, respectively. As shown in Fig. 1b, from 6 h to 24 h after seeding, cells were in logarithmic growth phase with or without PRRSV inoculation, and PRRSV infection showed little effect on cell proliferation, which was sustainable about 24 h or longer. However, the total cell number reduced greatly at 36, 48, and 72 h with an infective dose of 1 MOI. Because the total cell number decreased quickly between 24 and 36 h after infection with 5 MOI, 1 MOI was used in the following experiments. After infection with 1 MOI, MARC-145 cells showed typical cytopathic effects(CPE) and the CPE became stronger and stronger from 24 h to 48 h post-infection (Fig. 1c).

### PRRSV infection induces apoptosis in MARC-145 cells

**Fig. 2:**
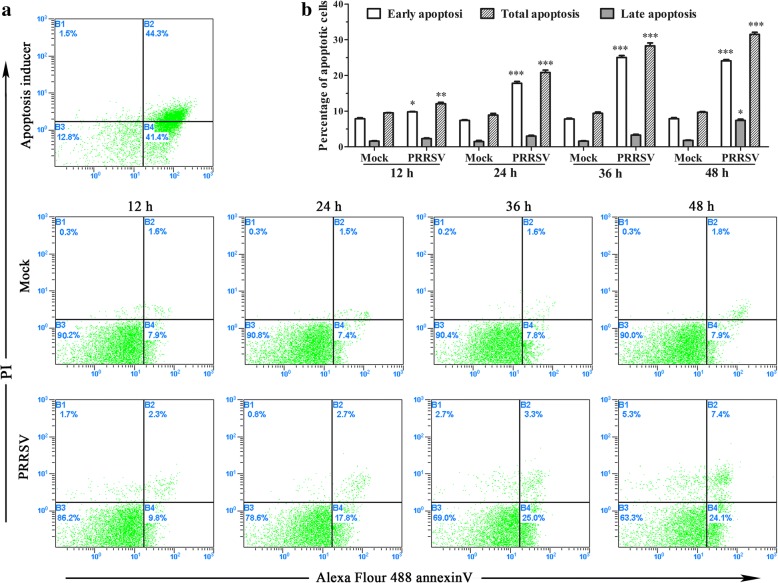
PRRSV infection induces apoptosis in MARC-145 cells. **a** Cell apoptosis was analyzed using annexin V/PI staining. MARC-145 cells were infected with 1 MOI PRRSV, and the apoptotic cells at 12, 24, 36, and 48 h post-infection were analyzed using Alexa Fluor® 488 annexin V/Dead cell apoptosis kit and flow cytometry. **b** Histogram represents the percentage of early and late apoptotic cells. Statistical anlysis is performed with GrapPad Prism version 6(GrapPad Software, Inc. Fay Avenue, CA, USA) using one-way analysis of variance(one-way ANOVA) followed by Turkey: compare all pairs of column. * indicates *p* < 0.05, ** indicates *p* < 0.01, and *** indicates *p* < 0.001. The following statistical method and differential representation method are the same

PRRSV infection can induce cell apoptosis both in vivo and in vitro. Cell apoptosis has been reported in alveolar macrophages, porcine intravascular monocytes, lymphocytes, and testicular germ cells of infected pigs which corresponds to a sharp reduction in these cell numbers in PRRSV positive swine [5, 12, 13]. We infected MARC-145 cells with PRRSV SD16 at 1 MOI and then examined cell apoptosis using an Alexa Fluor® 488 annexin V/Dead cell apoptosis kit. Our results show that the numbers of early and total apoptotic cells increased significantly after PRRSV infection. With the development of infection, more and more apoptotic cells were observed in MARC-145 cells infected with PRRSV when compared with those of mock-infected cells (Fig. 2a). At 48 h after PRRSV infection, the percentages of early and late apoptotic cells in PRRSV-infected MARC-145 cells were remarkably higher than those in mock-infected cells (24.1% ± 0.6% and 7.4% ± 0.6% versus 7.9% ± 0.5% and 1.8% ± 0.2%, respectively) (Fig. 2b).

### PRRSV infection leads to MARC-145 cell cycle arrest at G2/M phase

**Fig. 3:**
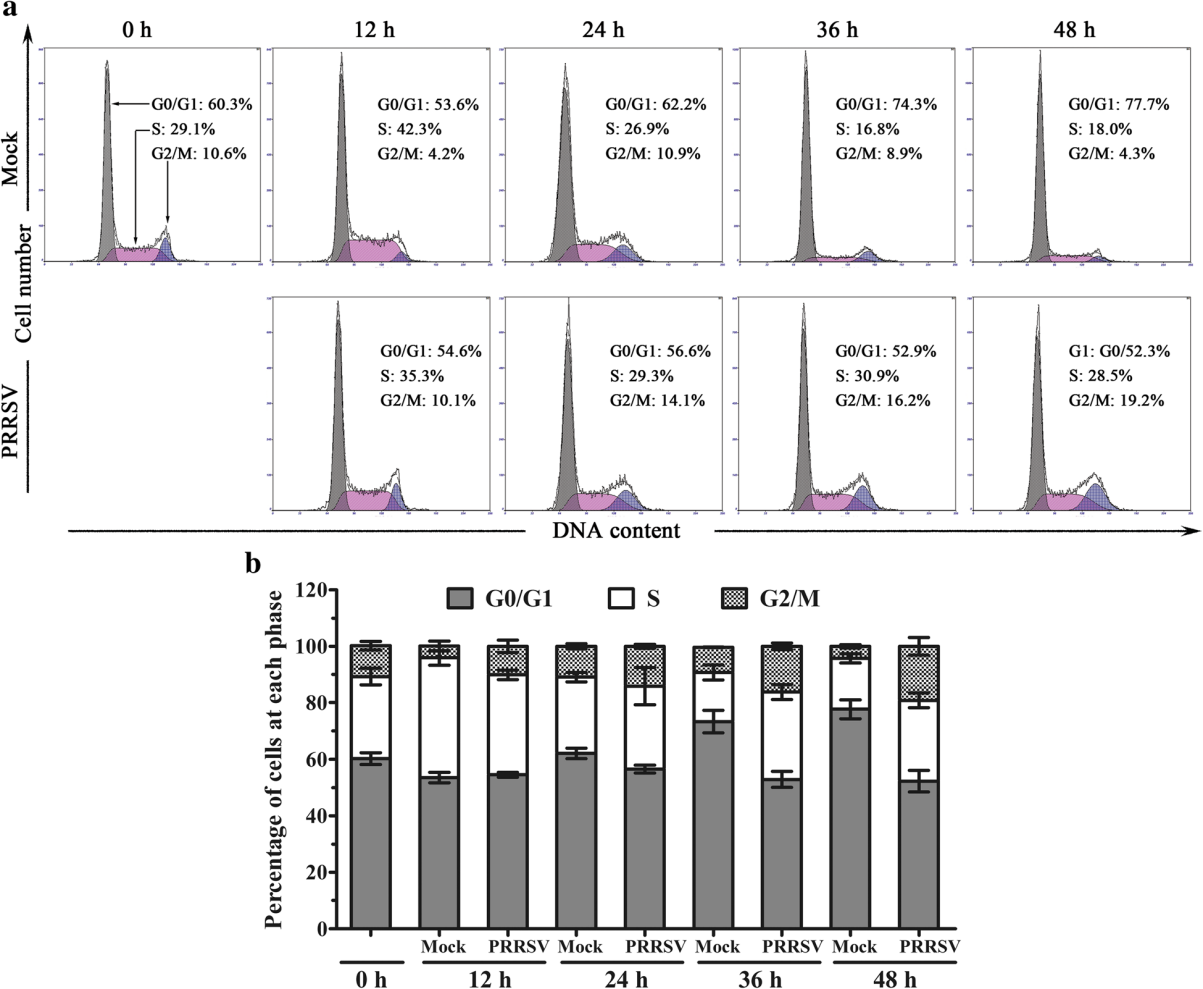
PRRSV infection leads to MARC-145 cells cycle arrest at G2/M phase. MARC-145 cells mock- or 1 MOI PRRSV-infected were collected, and cell cycle was analyzed by PI staining and flow cytometry. **a** The DNA contents of MARC-145 cells at 0, 12, 24, 36, and 48 h post-infection were determined by PI staining and analyzed by flow cytometry. **b** Histogram represents the percentages of mock- and PRRSV-infected MARC-145 cells in G0/G1, S, and G2/M phases. Data are mean ± SEM from three independent experiments

It is well known that many viruses can induce cell cycle arrest in various kinds of cells [10, 14]. The decrease in cell numbers in PRRSV-infected cells promptes us to determine whether PRRSV infection is associated with an arrest of cell division during a specific phase in the cell cycle in addition to inducing cell apoptosis. To address this question, cell cycle analysis of mock-infected and 1 MOI PRRSV-infected MARC-145 cells was performed at 0, 12, 24, 36, and 48 h post-infection by PI staining and flow cytometry. Representative cell cycle profiles and histograms of mock- and PRRSV-infected cells are presented in Fig. 3a and b, respectively. As shown in Fig. 3a, mock-infected MARC-145 cells maintained a normal cell cycle profile, and more than 75% cells were in G0/G1 phase when cells were in the state of contact inhibition after culture 48 h. However, PRRSV infection disturbed the normal cell cycle, some cells were arrested at G2/M phase and can not enter the next cell cycle, which resulted in the accumulation of cells in the G2/M phase. This phenomenon became more and more obvious with the development of virus infection. In addition, the percentages of PRRSV-infected cells in S phase also increased from 24 h to 48 h post infection and had a significant difference at 36 h and 48 h post-infection. At 48 h, the cells in G0/G1 phase decreased greatly, while cells in the G2/M phase increased significantly (Fig. 3b). These results demonstrate that PRRSV infection promoted the cycle progression of MARC-145 cells from G0/G1 phase to G2/M phase and then arrest in G2/M phase.

### Increased cyclinB1 levels in PRRSV-infected MARC-145 cells

**Fig. 4:**
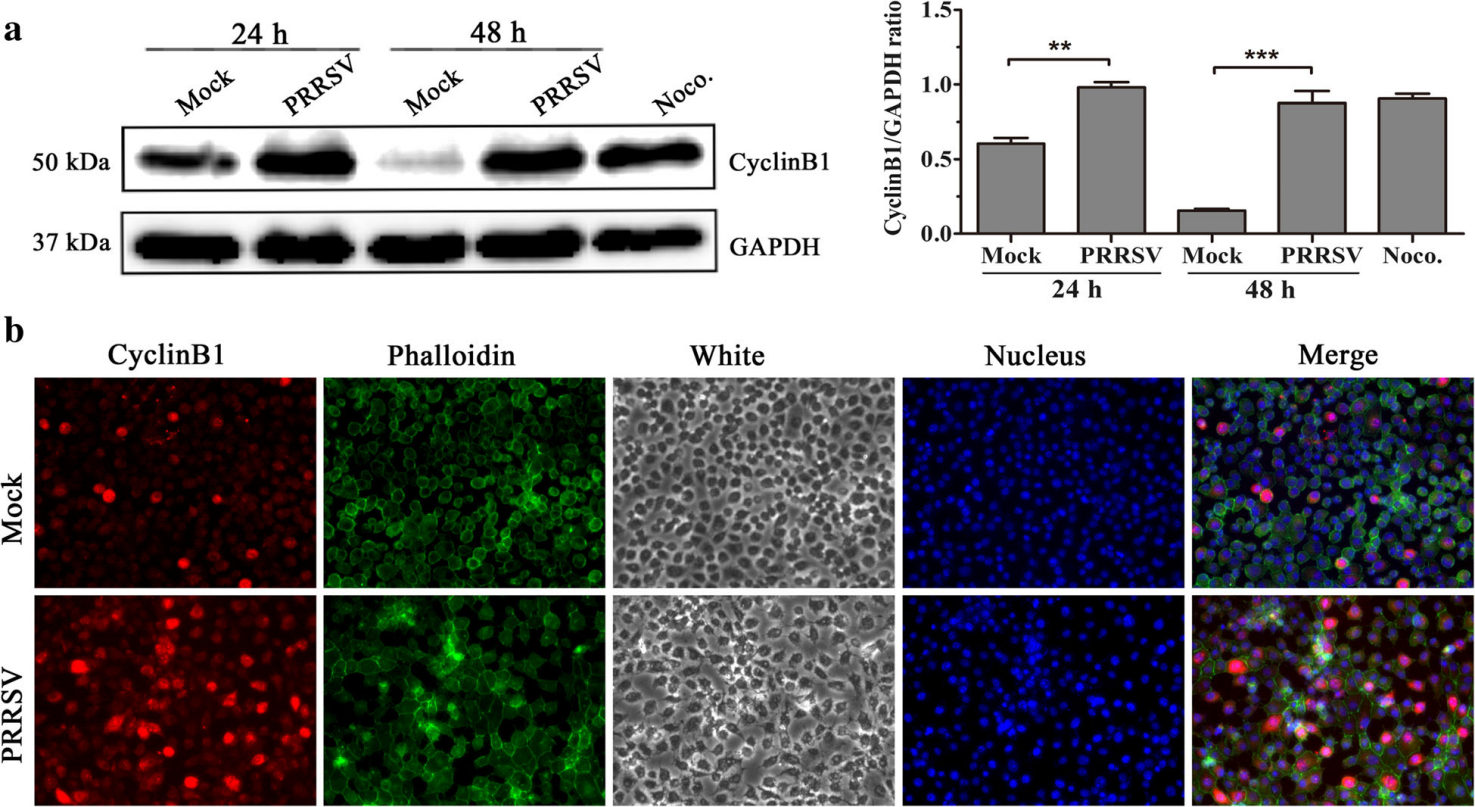
PRRSV infection induces increase of cyclinB1 expression. **a** Detection of cyclinB1 expression with western blot. MARC-145 cells mock-infected and 1 MOI PRRSV-infected were collected at 24 h and 48 h after PRRSV infection. CyclinB1 expression was detected with western blot using a specific antibody against cyclinB1. MARC-145 cells treated with 50 ng/mL nocodazole(Noco.) for 16 h served as a positive control (left), and expression levels were quantitatively analyzed and compared with GAPDH expression using Image J(https://imagej.nih.gov/ij/index.html) (right). ** indicates *p* < 0.01, *** indicates *p* < 0.001. **b** Detection of cyclinB1 expression and localization with IFA. Mock- and PRRSV-infected MARC-145 cells at 48 h post-infection were stained with an anti-cyclinB1 antibody, Phalloidin, and DAPI to determine cyclinB1(red), filamentous actin (F-actin) (green), and DNA (blue). Phalloidin(Phalloidin belongs to a class of toxins called phallotoxins. It functions by binding and stabilizing F-actin and effectively prevents the depolymerization of actin fibers. The properties of phalloidin make it a useful tool for investigating the distribution of F-actin in cells by labeling phalloidin with fluorescent analogs and using them to stain F-actin for light microscopy.) was used to show the outline of the cells. Then, the cells were visualized using Leica microsystems (Leica AF6000, Germany) (× 200)

The life cycle of a dividing cell can be split into four stages: G1, S, G2 and mitosis(M), with cells that are no longer cycling being said to be quiescent or in G0 phase. Progression from one stage to the next is controlled by the activities of kinase complexes made up of cyclins bound to cyclin-dependent kinases (Cdk). Mitosis is thought to be triggered by Cdk1 (also known as Cdc2 or p34^cdc2^ kinase) whose activation begins when it binds to its regulatory subunit cyclinB1. It accumulates in S and G2 phases to form a mitosis-promoting factor (MPF) with Cdc2 and is then ubiquitinated and degraded by the anaphase-promoting complex (APC) after the cells pass through mitosis [15]. To determine whether PRRSV infection affects cyclinB1 expression, the expression of cyclinB1 protein was detected by western blot at different times post-infection. Results showed that the expression of cyclinB1 in PRRSV infected MARC-145 cells increased significantly at 24 (*p* < 0.01) and 48 h (*p* < 0.001) post-infection compared with those of mock-infected cells (Fig. 4a and b). CyclinB1 shuttles between the nucleus and cytoplasm during interphase, and is known to be localized in the cytoplasm at the G2 phase and to be transported into the nucleus during the M phase [16]. Several reports have shown that virus infection-induced cell cycle arrest in G2 is due to the prevention of nuclear localization of cyclinB1 [17, 18]. In this study, although the expression of cyclinB1 was found in the cytoplasm and the nucleus in PRRSV-infected cells at 48 h, its expression was obviously higher than that in mock-infected cells. What’s more, in white light, the infected cells showed typtical CPE (Fig. 4b). These results clearly indicate that G2/M phase arrest induced by PRRSV infection does not result from loss of cyclinB1 or from interference with its nuclear translocation.

### PRRSV infection increases phosphorylated Cdc2(p-Cdc2) (Tyr15) expression in MARC-145 cells

**Fig. 5:**
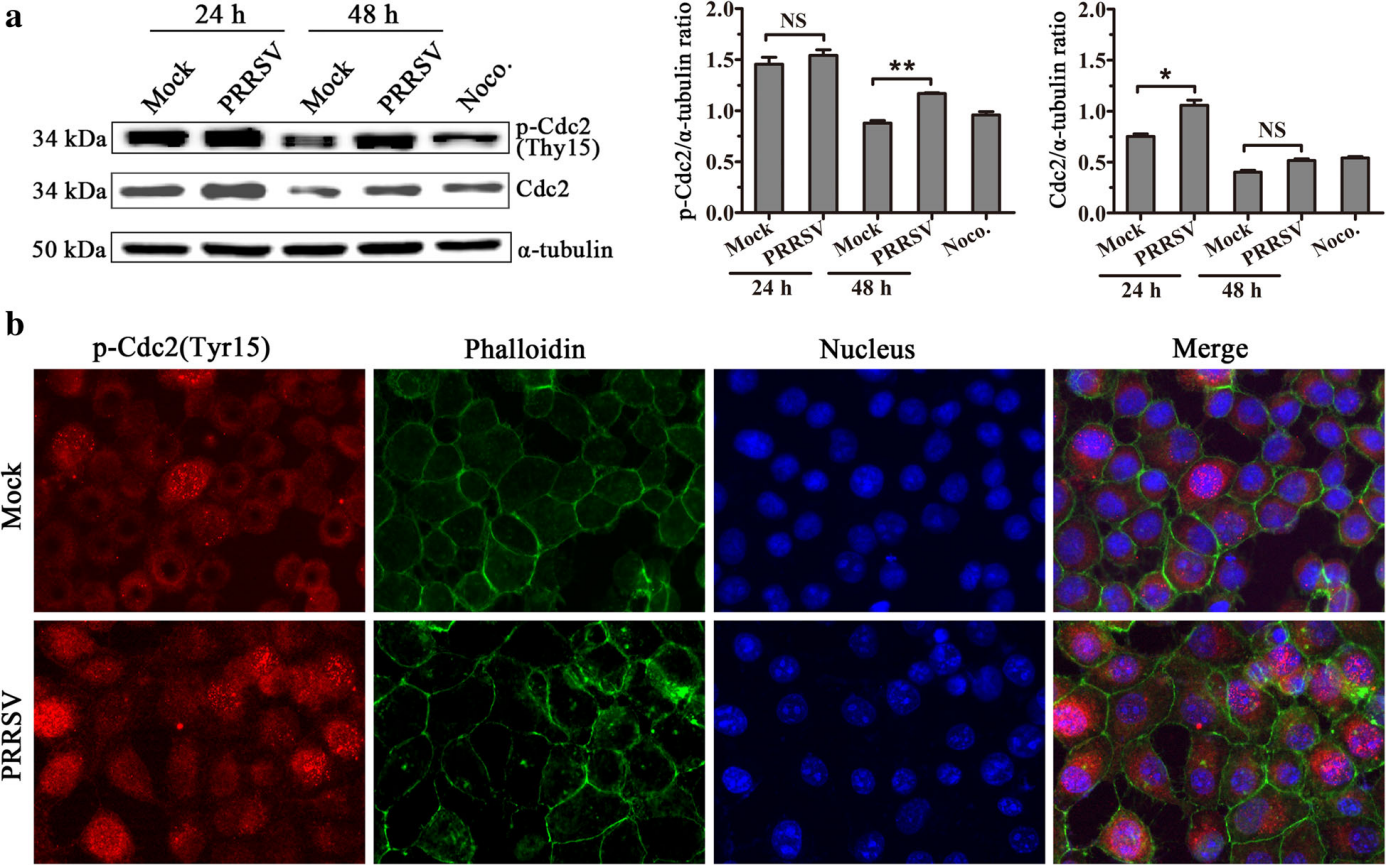
Phosphorylated Cdc2 (Tyr15) expression increases after PRRSV infection. **a** Detection of Cdc2 and p-Cdc2 (Tyr15) expression with western blot. MARC-145 cells mock-infected and 1 MOI PRRSV-infected were collected 24 h and 48 h after PRRSV infection. Cdc2 and p-Cdc2 (Tyr15) expression was detected with western blot using specific antibodies against Cdc2 or p-Cdc2 (Tyr15). MARC-145 cells treated with 50 ng/mL Noco. for 16 h served as a positive control (left), and their expression levels were quantitatively analyzed and compared with α-tubulin expression using Image J (right). * indicates *p < 0.05,* ** indicates *p < 0.01*. **b** Detection of p-Cdc2 (Tyr15) expression and localization with IFA. Mock- and PRRSV-infected MARC-145 cells at 48 h post-infection were stained with an anti-p-Cdc2 (Tyr15) antibody, Phalloidin, and DAPI to determine p-Cdc2 (Tyr15) (red), F-actin (green), and DNA (blue). Phalloidin was used to show the outline of the cells. Then, the cells were visualized using Leica microsystems (Leica AF6000, Germany) (× 630)

The Cdc2 kinase is the key regulator of the G2/M phase. Before mitosis, cyclinB-Cdc2 complexes are held in an inactive state by phosphorylation of Cdc2 at Thr14 and Tyr15, which is catalyzed by the protein kinases Wee1 (which phosphorylates Tyr15 only) and Myt1 (which phosphorylates both Thr14 and Tyr15). Cdc2 activation at the onset of mitosis results from the concurrent inhibition of Wee1 and Myt1 and stimulation of the phosphatase Cdc25C [15]. The increase in Tyr15 phosphorylation on Cdc2 is associated with multiple cell cycle arrests, especially G2/M phase arrest [19, 20]. To determine whether PRRSV infection also affects Cdc2 activity, the expressions of Cdc2 and p-Cdc2(Tyr15) were detected with western blot. Results revealed that the expression of Cdc2 in PRRSV-infected cells was higher than that in mock-infected cells at 24 h and the expression of p-Cdc2(Tyr15) in PRRSV-infected cells was significantly higher than that in mock-infected cells at 48 h post-infection (Fig. 5a), which indicated the G2/M phase arrest caused by PRRSV infection is related with expression increase of p-Cdc2(Tyr15). The increase of p-Cdc2(Tyr15) and its distribution in PRRSV-infected MARC-145 cells at 48 h post-infection was further confirmed by immunofluorescence analysis (Fig. 5b).

### Effects of PRRSV infection on G2/M cell cycle-regulatory proteins Wee1, Myt1, Cdc25C, p-Cdc25C, and Chk1

**Fig. 6:**
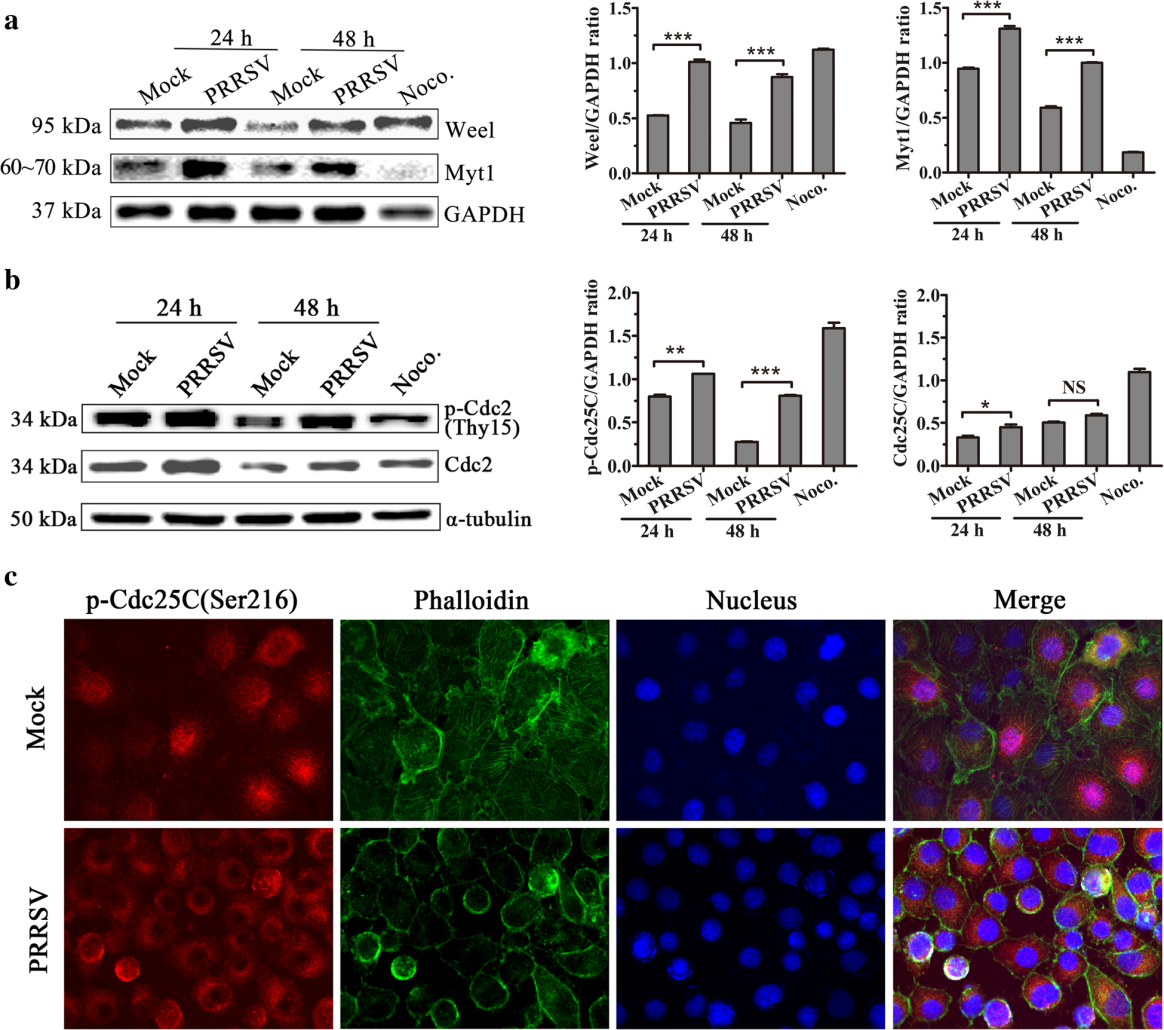
Wee1 and Myt1 expression and Cdc25C phosphorylation enhance after PRRSV infection. **a** Wee1 and Myt1 expression in mock-and PRRSV-infected MARC-145 cells. Cell lysates were collected at the indicated time points post-infection, and the expression of Wee1 and Myt1 was determined by western blot. MARC-145 cells treated with 50 ng/mL Noco. for 16 h served as a positive control (left). Wee1 and Myt1 expression levels were quantitatively analyzed and compared with GAPDH expression level using Image J (right). *** indicates *p* < 0.001. **b** PRRSV infection induced phosphorylation of Cdc25C in infected MARC-145 cells. Lysates from mock-and PRRSV-infected cells were prepared at the indicated time points and processed for western blot with specific antibodies against Cdc25C and phospho-Cdc25C (Ser216). MARC-145 cells treated with 50 ng/mL Noco. for 16 h served as a positive control (left). Phosphorylated Cdc25C and Cdc25C protein levels were quantitatively analyzed and compared with GAPDH expression levels using Image J(right). * indicates *p < 0.05,* ** indicates *p < 0.01*, *** indicates *p < 0.001*. **c** Cytoplasmic accumulation of p-Cdc25C (Ser216) in PRRSV-infected MARC-145 cells. Mock- and PRRSV-infected MARC-145 cells at 48 h post-infection were stained for p-Cdc25C (Ser216) (red), F-actin (green), and DNA (blue) with an anti-p-Cdc25C (Ser216) antibody, Phalloidin, and DAPI. Then, the cells were visualized using Leica microsystems (Leica AF6000, Germany) (× 630)

Considering that Cdc2 kinase activity is negatively regulated by kinases Wee1 and Myt1 and positively regulated by phosphatase Cdc25C, we further investigated the expression of these G2/M cell cycle-regulatory proteins during PRRSV infection. As shown in Fig. 6a, Wee1 expression levels were higher in PRRSV-infected cells than those in mock-infected cells at 24 h(1.728-fold) and 48 h(1.885-fold) post-infection, and Myt1 expression levels were also higher in PRRSV-infected cells than those in mock-infected cells at 24 h(1.249-fold) and 48 h(1.6635-fold) post-infection. These data suggest that Wee1 and Myt1 are involved in the regulation of G2/M arrest induced by PRRSV infection. In addition, we found that Wee1 expression in nocodazole-treated cells also increased greatly, but Myt1 expression was hardly detected, which may be a result of protein degradation during M phase.

Cdc25C is a Cdc2-specific phosphatase. Studies have suggested that phosphorylation of Cdc25C on Ser216 by Chk1 or Chk2 leads to 14–3-3 protein binding, resulting in the sequestration of Cdc25C in the cytoplasm and cytoplasmic accumulation of phospho-Cdc25C (Ser216) denies access to its substrate Cdc2 subunit and prevents cells from going into mitosis by keeping the MPF inactive, resulting in the arrest of cells at G2/M [21]. We analyzed the expression of Cdc25C and phosphorylated Cdc25C(p-Cdc25C). As displayed in Fig. 6b, PRRSV-infected cells showed increased levels of the Ser216-phosphorylated form of Cdc25C compared with those in mock-infected controls at 24 and 48 h post-infection. The expression and distribution of p-Cdc25C(Ser216) was also analyzed using IFA (Fig. 6c), p-Cdc25C(Ser216) expression was obviously detectable and mainly located in the cytoplasm of PRRSV-infected cells, in contrast to its expression in mock-infected cells. These results collectively suggest that PRRSV infection gives rise to the level of Cdc2 phosphorylation increase in PRRSV-infected cells by enhancing Wee1 and Myt1 expression and prevents Cdc2 dephosphorylation by inhibiting Cdc25C activity, which inhibits of the activity of Cdc2 in infected cells and results in the arrest of cells at G2/M phase.

**Fig. 7:**
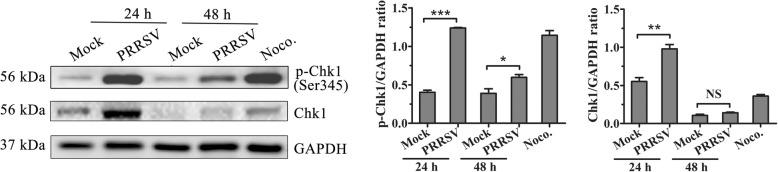
PRRSV infection significantly induces Chk1 expression and phosphorylation of Chk1. Lysates from mock- or PRRSV-infected MARC-145 cells were prepared at the indicated time points and were processed for western blot with specific antibodies against Chk1 and phospho-Chk1 (Ser345). MARC-145 cells treated with 50 ng/mL Noco. for 16 h served as a positive control (left). Phosphorylated Chk1 and Chk1 protein levels were quantitatively analyzed and compared with GAPDH expression levels using Image J(right). * indicates *p < 0.05,* ** indicates *p < 0.01*, *** indicates *p < 0.001*

The DNA damage checkpoint kinases, Chk1 and Chk2, play important roles in regulating the G2/M checkpoint via the phosphorylation of Cdc25C at Ser216 through an ATM/ATR-dependent pathway [22, 23] . Given that we have found increased p-Cdc25C(Ser216) levels in PRRSV-infected cells, we further analyzed Chk1 activation with western blot. As expected, PRRSV infection significantly enhanced Chk1 activation by increasing phosphorylation of Chk1 at Ser345 in PRRSV infected cells (Fig. 7).

### PRRSV infection results in activation of p53/p21 signaling pathway

**Fig. 8:**
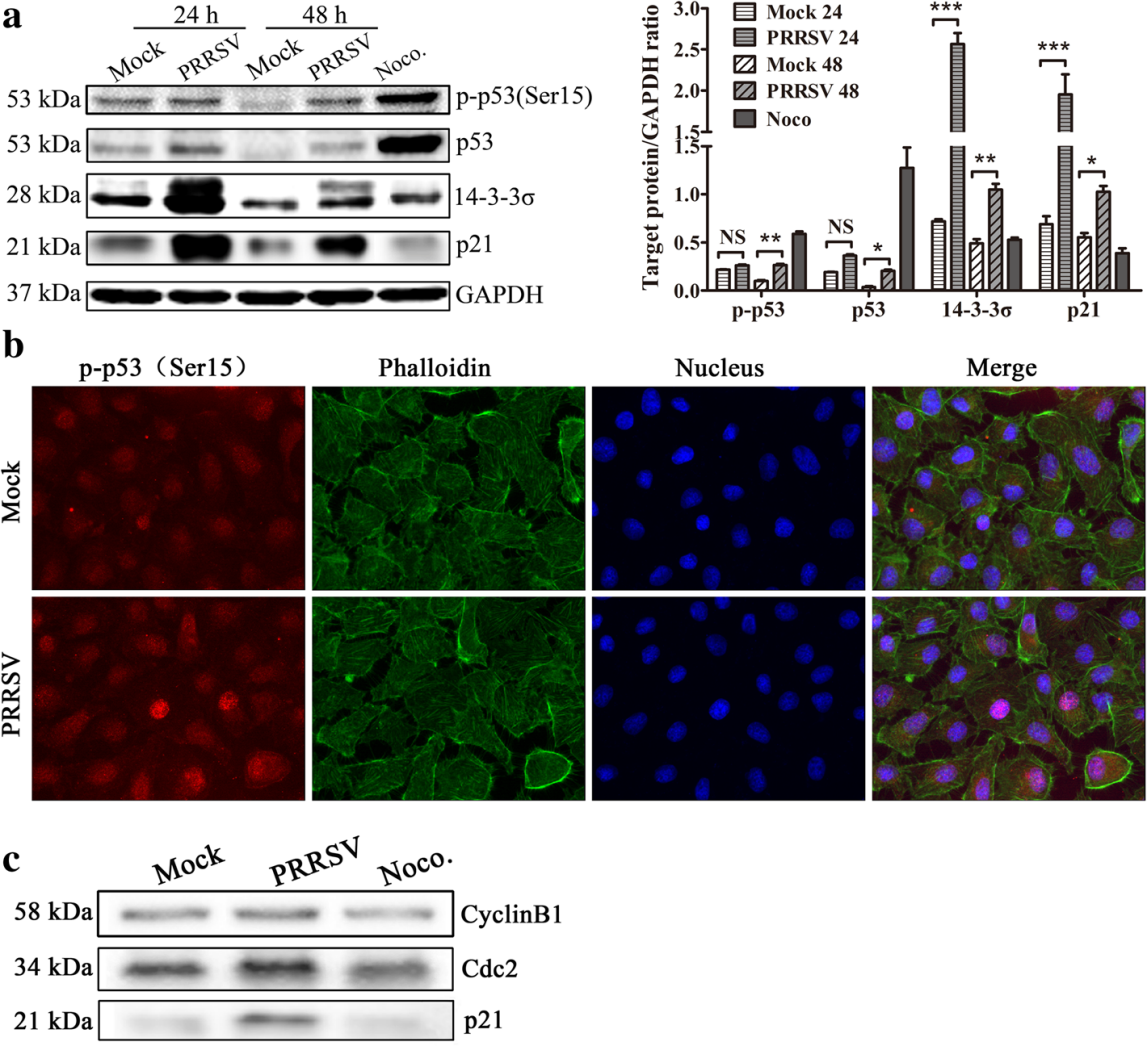
Expression and/or phosphorylation of several cell cycle checkpoint proteins in PRRSV-infected MARC-145 cells. **a** PRRSV infection markedly induced the expression of p53, p-p53, 14–3-3σ, and p21 in MARC-145 cells. Cell lysates were prepared, and the expression of p53, p-p53, 14–3-3σ, and p21 was determined with western blot. MARC-145 cells treated with 50 ng/mL Noco. for 16 h served as a positive control (left). Targeted protein expression levels were quantitatively analyzed and compared with GAPDH expression levels using of Image J (right). * indicates *p < 0.05*, ** indicates *p < 0.01*, *** indicates *p < 0.001*. **b** p-p53(Ser15) expression in MARC-145 cells was visualized using IFA. PRRSV- and mock-infected cells were stained for p-p53(Ser15) (red), F-actin (green), and DNA (blue) with p-p53(Ser15) antibody, Phalloidin, and DAPI stain at 48 h post-infection. Then, the cells were visualized using Leica microsystems (Leica AF6000, Germany) (× 630). **c** Interactions between p21 and Cdc2-cyclinB1 in MARC-145 cells induced by PRRSV infection. Dynabeads-Ab complex was prepared by incubating Cdc2 mouse mAb with Protein G Dynabeads using a Catch and Release(version 2.0) reversible immunoprecipitation system (ThermoFisher, USA). Then, the supernatants of mock-infected, PRRSV-infected, or nocodazole-treated cells lysis were added to the tubes containing Dynabeads-Ab complex and incubated overnight at 4 °C. After washing with PBS, p21^Walf1/Cip1^ rabbit mAb and cyclinB1 antibody were used to detect the Dynabeads®-Ab-Ag complex with western blot

p53 is a transcription factor that is induced in response to DNA damage and/or cellular stress, which controls the G2/M checkpoint by allowing sufficient repairs to occur before the cell enters mitosis [24]. Ser15 phosphorylation of p53(Ser18 phosphorylation in mice) can lead to stability increase of p53, a common event in DNA damage and other stress responses [25, 26]. Phosphorylation of p53 usually correlates with the ability of p53 to transactivate a number of downstream genes to mediate either cell cycle arrest or apoptosis. p21 is a cyclin-dependent kinase inhibitor located in the downstream of the p53 gene that can inhibit the activity of the Cdc2-cyclinB1 complex. p53 also regulates the G2/M checkpoint through induction of 14–3-3 sigma(σ), a protein that protects damaged cells from entry into mitosis by binding and sequestering Cdc2-cyclinB1 complexes in the cytoplasm [27]. To investigate the relationship between G2/M arrest induced by PRRSV infection and the p53 signaling pathway, we examined the expressions of p53, p-p53(Ser15), 14–3-3σ, and p21 using western blot and p-p53(Ser15) with IFA. The results show that the expression of 14–3-3σ and p21 increased significantly at 24 and 48 h after PRRSV infection, while p-p53(Ser15) and p53 expression was only upregulated at 48 h after PRRSV infection (Fig. 8a and b). This indicates that the cell cycle G2/M arrest caused by PRRSV infection is also associated with p53 signal pathway.

We further conducted immunoprecipitation assay using Cdc2 antibody to precipitate p21. The result confirms the interaction between p21 and Cdc2-cyclinB1 in MARC-145 cells infected by PRRSV (Fig. 8c). These results reveal that activation of the p53/p21 signaling pathway may also be one reason for G2/M arrest of PRRSV-infected cells.

### PRRSV 1 and 2 strains induce cyclinB1 and p-Cdc2 (Tyr15) expression increase

**Fig. 9:**
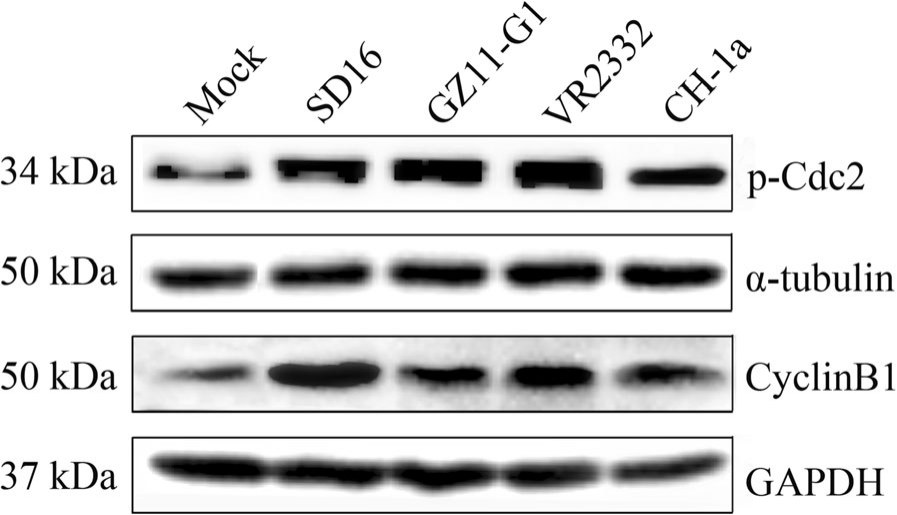
PRRSV 1 and 2 strains infection leads to expression increase of cyclinB1 and p-Cdc2(Tyr15). MARC-145 cells mock-infected and 1 MOI different PRRSV strains-infected were collected at 48 h post-infection. CyclinB1 expression and p-Cdc2(Tyr15) were detected with western blot using specific antibody

To determine whether different PRRSV strains can induce MARC-145 cell cycle arrest, we used PRRSV 2 strains SD16, VR2332, CH-1a and PRRSV 1 strain GZ11-G1 infected MARC-145 cells. At 48 h post-infection, cells were collected and cyclinB1 and p-Cdc2 (Tyr15) expression were detected with western blot. As expected, PRRSV 1 and PRRSV 2 strains infection all induces cyclinB1 and p-Cdc2(Tyr15) expression increase, which indicates that PRRSV induces MARC-145 cell cycle arrest is common (Fig. 9).

## Discussion

PRRSV, a globally dangerous pathogen in the swine industry, has raised heightened concerns with the emergence of its highly pathogenic viral form and difficulties in prevention and treatment. Primary PAMs are the major target of PRRSV infection and are the best cell model for studying PRRSV biology. However, PAM is a terminally differentiated cell and can not divide and proliferate. In vitro, PRRSV also can be propagated in epithelial-derived MARC-145 cells, a subclone of the African green monkey kidney cell line MA104. Manipulation of the cell cycle in infected cells is a common strategy used by many viruses to regulate and amplify their infection. In this study, we demonstrated that PRRSV infection promoted MARC-145 cell cycle arrest in G2/M phase, which may be one of the key mechanisms responsible for PRRSV-induced immunosuppression in infected hosts.

The G2/M DNA damage checkpoint serves to prevent the cell from entering mitosis(M phase) with genomic DNA damage. The activity of the Cdc2-cyclinB1 complex is pivotal in regulating the G2-phase transition, wherein Cdc2 is maintained in an inactive state by the tyrosine kinases Wee1 and Myt1. DNA damage cues activate the sensory DNA-PK/ATM/ATR kinases, which relay two parallel cascades that ultimately serve to inactivate the Cdc2-cyclinB1 complex. The first cascade rapidly inhibits progression into mitosis: the Chk kinases phosphorylate and inactivate Cdc25, preventing Cdc2 activation. The slower second parallel cascade involves the phosphorylation of p53 and allows for its dissociation from MDM2 and MDM4, which activates DNA binding and transcriptional regulatory activity, respectively. The second cascade constitutes the p53 downstream-regulated genes including: 14–3-3 that bind to the phosphorylated Cdc2-cyclinB1 complex and exports it from the nucleus; GADD45, which binds to and dissociates the Cdc2-cyclinB1 complex; and p21, an inhibitor of a subset of the cyclin-dependent kinases that includes Cdc2.

CyclinB1 is an important regulatory factor in the normal cell cycle process. Its expression has periodic behavior that is parallel to the expression of MPF activity. During interphase, the concentration of cyclinB1 gradually increases following G1, S, and G2 phases and reaches a critical threshold at the end of G2. At the threshold, Cdc2 activation occurs and triggers the onset of mitosis, then cyclinB1 is degraded after the cells pass through mitosis [28]. While cyclinB1 shuttles between the nucleus and cytoplasm during the interval, it is known to be localized in the cytoplasm at G2 phase and to be transported into the nucleus during M phase [16]. To explore the mechanisms responsible for the promotion of cell cycle arrest by PRRSV, we first analyzed the expression and location of cyclinB1 after PRRSV infection. We found that it significantly accumulates in PRRSV-infected cells compared with that of mock-infected control (Fig. 4a), and although the expression of cyclinB1 was found in the cytoplasm and the nucleus in PRRSV-infected cells at 48 h, its expression was obviously higher than that in mock-infected cells (Fig. 4b). This reveals that PRRSV infection does cause cell cycle arrest.

The Cdc2 kinase encoded by the Cdc2 gene is a cyclin-dependent-kinase that specifically regulates the G2/M phase and interacts primarily with cyclinB1 to regulate G2/M transition. The activity of Cdc2 is also regulated by phosphorylation, dephosphorylation, and changes in its subcellular localization. When the cells enter the G2 phase, Thr14 and Tyr15 on Cdc2 are dephosphorylated, then cyclinB1 and Cdc2 combine into an active molecule and participate in the regulation of G/M checkpoint. The increase in Tyr15 phosphorylation on Cdc2 is associated with multiple cell cycle arrests, especially the G2/M phase arrest [29]. Myt1 is a cell membrane-associated protein kinase that is able to bind and phosphorylate Cdc2 at both Thr14 and Tyr15, preventing its nuclear translocation [30]. Wee1 suppresses Cdc2 kinase activity by phosphorylation at Tyr15 in the nucleus [31]. By the end of G2 phase, Myt1 and Wee1 are inactivated, and a specific dual-phosphatase, Cdc25, is activated. Activated Cdc25 dephosphorylates two residues(Thr14 and Tyr15) in Cdc2, leading to activation of Cdc2. After PRRSV infection, phosphorylated Cdc2 at Tyr15, Myt1, Weel expression (Fig. 5a and Fig. 6a), and phosphorylated Cdc2 nuclear distribution(Fig. 5b) all increased, indicating that Cdc2 activity is inhibited and leads to the infected cell cycle arrest at the G2/M phase.

We further focused on Cdc25C expression and distribution after PRRSV infection. Cdc25C dephosphorylates the Thr14 and Tyr15 residue of Cdc2 and triggers entry into mitosis [32]. When Cdc25C activity is inhibited, the activity of Cdc2-cyclinB1 complex will also be inhibited, the total switch of the G2 checkpoint is in the “off” state, and the G2/M phase block occurs [32]. Throughout interphase, human Cdc25C is phosphorylated on serine 216 and bound to 14–3-3 proteins, resulting in the sequestration of Cdc25C in the cytoplasm. Cytoplasmic accumulation of phospho-Cdc25C(Ser216) denies access to its substrate Cdc2 subunit and prevents cells from going into mitosis by keeping the MPF inactive, resulting in the arrest of cells at the G2/M phase. Dephosphorylation of S216 and dissociation of 14–3-3 from Cdc25C is one of the events required for the initiation of mitosis [18, 21]. Although increase of Cdc25C expression in PRRSV-infected cells was not obvious at 48 h, the Ser216-phosphorylated form of Cdc25C in PRRSV-infected cells showed remarkable increase compared with those in mock-infected controls at 24 and 48 h post-infection (Fig. 6b). In contrast with mock-infected cells, phospho-Cdc25C(Ser216) expression was obviously detectable and distributed mainly in the cytoplasm of PRRSV-infected cells(Fig. 6c). This further suggests that PRRSV infection can inhibit the Cdc2-dependent phosphatase Cdc25C and result in the inhibition of Cdc2 activity.

The DNA damage checkpoint kinases, Chk1 and Chk2, are in the upstream of Cdc25. Chk1-mediated phosphorylation of Cdc25C at Ser216 promotes its degradation and abolishes the activation of Cdc2-cyclinB1 kinases, thereby causing G2/M arrest [33]. Chk1 kinase activity is rapidly stimulated in a cell cycle phase-specific manner in response to both DNA damage and replication arrest. The extent and duration of activation correlates closely with regulatory phosphorylation at S317, S345, and S366, where S345 phosphorylation relieves the repression of latent Chk1 catalytic activity through checkpoint activation [34]. Compared to the phosphorylation of Chk2 and Chk1 at 72 h post-infection in HHV-6A-infected HSB-2 cells [18], Chk1 and phospho-Chk1(Ser345) expression all increased significantly at 24 h after PRRSV infection. At 48 h post-infection, phospho-Chk1(Ser345) expression was still significantly elevated. This suggests that different viruses elicit the ATM/ATR DNA damage checkpoint signaling pathway at different stages.

As a crucial cell cycle regulator, the p53 tumor suppressor has an important role in the cellular response to various agents by transcriptionally activating numerous genes involved in DNA repair and cell cycle arrest. The p53-dependent arrest of cells at the G1/S or G2/M phase is an important component of the cellular response to genotoxic stress, including viral infection [35, 36]. The first transcriptional target of p53 is p21, a CKI of the Cip/Kip family, which bridges the function of p53 with the cell cycle and plays important roles in regulating cell cycle progression or arrest. Four mechanisms have been postulated for how p21 participates in inhibiting Cdc2 activity to cause G2 arrest. First, p21 inhibits Cdk activity by binding directly to Cdk/Cyclin complexes. In the second mechanism, p21 causes loss of Cdc2 activity by inhibiting Cdk2. The third mechanism is p21 can interfere with the activating phosphorylation of Cdc2 by CAK. The fourth mechanism depends on the fact that p21 binds to PCNA, a processivity factor for DNA polymerases δ and ε that is required for DNA synthesis and repair [37]. In this study, the interaction between p21 and Cdc2-cyclinB1 complex in MARC-145 cells infected by PRRSV was confirmed with immunoprecipitation assay using Cdc2 antibody to precipitate p21 (Fig. 8c), which indicating that p21 uses the first mechanism participating in inhibiting Cdc2 activity to cause G2 arrest in PRRSV infected MARC-145 cells. Our study showed that the p53/p21 pathway is also involved in the G2/M cell cycle arrest of PRRSV-infected MARC-145 cells, where PRRSV infection increased the expression and phosphorylation of p53. The activation of p53 resulted in p21 expression and the subsequent binding of p21 protein to the Cdc2-cyclinB1 complex, which inhibited the activity of the complex and blocked the G2/M transition (Fig. 8a and c).

The protein 14–3-3σ, which can bind to and sequester Cdc2-cyclinB1 in the cytoplasm, is also a direct transcriptional target of p53. Overexpression of 14–3-3σ in HCT116 cells, using a recombinant adenovirus, caused most cells to arrest in G2 phase, where 14–3-3σ controlled the duration of G2 arrest in response to DNA damage in the epithelial colorectal tumor cell line HCT116 [27]. The western blot result shows that the expression of 14–3-3σ in PRRSV-infected MARC-145 cells was significantly increased at 24 h(*p < 0.001*) and 48 h (*p < 0.01*) compared with normal control cells (Fig. 8a), which clearly suggests that 14–3-3σ was also involved in the mechanism of the G2/M arrest caused by PRRSV.

We further infected MARC-145 cells using PRRSV 2 strains, VR2332 and CH-1a, PRRSV 1 strain, GZ11-G1, and analyzed cyclinB1 and p-Cdc2(Tyr15) expression (Fig. 9). The target proteins’ expression increased obviously in PRRSV infected groups compared with mock group which implied that cell cycle arrest at G2/M induced by PRRSV is not strain specific. Of course, this requires further tested with more different strains.

**Fig. 10:**
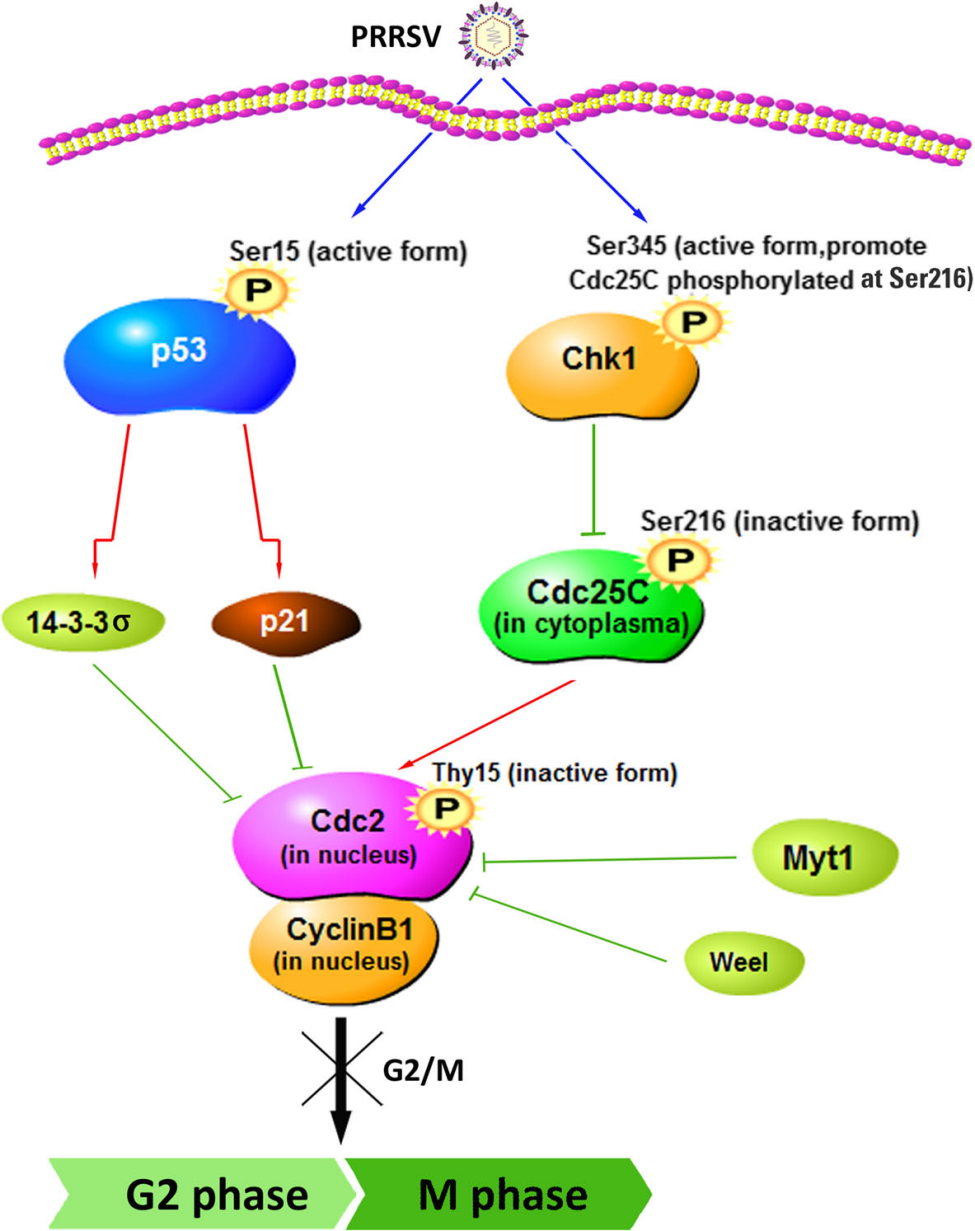
Proposed mechanisms of PRRSV-induced G2/M cell cycle arrest in MARC-145 cells. One the one hand, PRRSV infection activates Chk1, leading to cytoplasmic accumulation of Cdc25C and increasing of Cdc25C phosphorylation(Ser216) which is an inhibitory site of Cdc25C, therefore results in inhibition of Cdc25C activity. Inactivated Cdc25C and increased Wee1 and Myt1 expression promote downstream Cdc2 inhibitory phosphorylation(Tyr15) in the nucleus and consequently reduces the activity Cdc2-cyclinB1 complex. On the other hand, PRRSV infection activates p53/p21 signaling pathway which also inhibits the activity of Cdc2-cyclinB1 complex. The activity inhibition of Cdc2-cyclinB1 complex leads to G2/M cell cycle arrest in MARC-145 cells

The present study has suggested that PRRSV infection is able to regulate several key cellular regulatory proteins and resulted in G2/M cell cycle arrest (Fig. 10). Increasing evidences suggest that viruses interact with the host cell division cycle to create an optimal environment for their survival and/or replication [10, 35, 38]. Experiments with small molecule inhibitors have shown that arrest at G2/M phase can benefit the early stages of HIV life cycle by increasing the number of integrated proviruses [39]. Human enterovirus 68 (EV-D68) can manipulate the host cell cycle to arrest cells in G0/G1 phase, thus providing favorable conditions for virus production [38]. Our studies suggested that PRRSV, like other viruses, may have evolved mechanisms to alter the physiology of the host cells during viral infection in a manner beneficial to viral replication and pathogenesis. In fact, synchronization MARC-145 cells in the G2/M phase, not in the G0/G1 or S phase, promotes PRRSV production(data are not shown). It is possible that cell cycle arrest due to PRRSV infection prevents early death of infected cells, therefore allowing them to gain sufficient time and resources for (re)production.

## Conclusions

In conclusion, we show that PRRSV infection arrests cells in G2/M phase by activation of the Chk/Cdc25C and p53/p21 pathway. The G2/M phase delay is accompanied by an accumulation of cyclinB1 and increased p-Cdc2(Tyr15)-cyclinB1 complex formation, which is thus, advantageous for viral genome production and formation of new viral particles.

## Abbreviations

CCK-8: Cell counting kit-8
Cdc: cell division cycle
CPE: cytopathic effects
DMEM: Dulbecco’s modified eagle medium
FBS: fetal bovine serum
MOI: multiplicity of infection
MPF: mitosis-promoting factor
N: nucleocapsid
Noco.: Nocodazole
PAM: porcine alveolar macrophages
PBS: phosphate-buffered saline
p-Cdc2: phosphorylated Cdc2
PRRSV: Porcine reproductive and respiratory syndrome virus
